# Obituary

**Published:** 1921-05

**Authors:** 


### OBITUARY

### Dr. Robert A. Molyneaux

Doctor Molyneaux was born in Point Pleasant, Ohio, May 22, 1828, and died in Oxford, Ohio, March 28, 1921. He lived as a neighbor during his early life with U. S. Grant. He studied dentistry with Dr. George W. Keeley and graduated from the Ohio College of Dental Surgery in March, 1862. He practiced in New Richmond, Ohio, until 1907, when he retired and moved to Oxford, Ohio, where he died. He was married to Susan E. Kumler in 1862. Dr. Molyneaux had four children living.

Doctor Molyneaux was a man of most pleasing personality—refined, gentle, courteous and lovable. In his profession he stood high; among his fellowmen he was esteemed for his unquestionable integrity and moral worth. Early in life he united with the Presbyterian church, and for over fifty years, up to the time of his death, he was an elder in the church. His life, though simple, was an inspiration; his exemplary living was well worthy of emulation. During the years of his activity he was extremely charitable; he never was known to turn a deaf ear to a worthy appeal.

